# Complete Genome Sequence of *Mycoplasma mycoides* subsp. *mycoides* T1/44, a Vaccine Strain against Contagious Bovine Pleuropneumonia

**DOI:** 10.1128/genomeA.00263-16

**Published:** 2016-04-14

**Authors:** Géraldine Gourgues, Aurélien Barré, Emmanuel Beaudoing, Johann Weber, Ghislaine Magdelenat, Valérie Barbe, Elise Schieck, Joerg Jores, Sanjay Vashee, Alain Blanchard, Carole Lartigue, Pascal Sirand-Pugnet

**Affiliations:** a Institut national de recherche agronomique, UMR 1332 de Biologie du Fruit et Pathologie, Villenave d’Ornon, France; b Université de Bordeaux, UMR 1332 de Biologie du Fruit et Pathologie, Villenave d’Ornon, France; c Université de Bordeaux, Centre de bioinformatique et de génomique fonctionnelle, CBiB, Bordeaux, France; d Lausanne Genomic Technologies Facility, Center for Integrative Genomics, University of Lausanne, Lausanne, Switzerland; e Laboratoire de Biologie Moléculaire pour l’Etude des Génomes, (LBioMEG), CEA-IG-Genoscope, Evry, France; f International Livestock Research Institute (ILRI), Nairobi, Kenya; g Institute of Veterinary Bacteriology, University of Bern, Bern, Switzerland; h The J. Craig Venter Institute, Rockville, Maryland, USA

## Abstract

*Mycoplasma mycoides* subsp. *mycoides* is the etiologic agent of contagious bovine pleuropneumonia. We report here the complete genome sequence of the strain T1/44, which is widely used as a live vaccine in Africa.

## GENOME ANNOUNCEMENT

Contagious bovine pleuropneumonia (CBPP) is considered by the World Organization for Animal Health as one of the most severe animal diseases affecting cattle in sub-Saharan Africa. As a consequence, CBPP has a major impact on livestock-dependent people, causing reduced food supply and significant income losses because of trade restrictions. Several vaccine strains have been developed so far from the causative agent *Mycoplasma mycoides* subsp. *mycoides*, including strain T1/44, which is currently used in Africa. This attenuated strain was derived in the 1950s from a Tanzanian strain by 44 passages on embryonated eggs (1). The T1/44 strain provides partial protection to the vaccinated animals. However, it can still cause disease when applied directly into the lungs (2), indicating a retained virulence potential.

Here, we present the complete genome sequence of this important veterinary live vaccine strain. The culture used for gDNA extraction was the CIRAD-EMVT/PANVAC CBPP vaccine strain T1/44/2 (batch EMVT 002, June 1996), as stored in the collection maintained at Anses, Lyon’s laboratory, under the reference number 11278. This strain was grown at 37°C as previously described (3), and gDNA was extracted with the QiagenMagAttractHMW kit (100 to 200 kb) and quality-controlled by gel electrophoresis and a quantification by fluorescence.

The T1/44 genome was sequenced on a PacBio RSII sequencer at the Lausanne Genomic Technologies Facility. Its genome was assembled into one single contig using the HGAP version 3.0 (4) and toAmos (5) assembly software. Genome annotation was performed using Prokka (6) and further improved manually after integration in the MolliGen database (http://www.molligen.org) (7).

The genome of *Mycoplasma mycoides* subsp. *mycoides* T1/44 consists of a 1,188,848-bp chromosome with a G+C content of 23.92% and encoded 1,112 coding sequences, 2 rRNA sets, and 30 tRNAs. Its genome organization is very similar to the previously sequenced reference strain PG1 (8) (GenBank accession no. BX293980), except for an inversion of 173 kb between two IS-rich loci. This remarkable inversion (positions 1,173,545 to 1,188,848 and 1 to 157,822) includes the predicted chromosomal replication origin and was confirmed by optical mapping using the Argus system (OpGen) and PvuII as restriction enzyme. Interestingly, the T1-specific PCR assay developed previously by others is based on two primers, MmmSCP1 and T1M2, that are complementary to each side of the upstream junction. Therefore, the T1/44 genome sequence provides the explanation for the specificity of this assay with the ability to distinguish T1 strains from other vaccine strains (9).

### Nucleotide sequence accession number.

The annotated genome sequence was deposited in GenBank under accession number CP014346.
